# Online Denoising Based on the Second-Order Adaptive Statistics Model

**DOI:** 10.3390/s17071668

**Published:** 2017-07-20

**Authors:** Sheng-Lun Yi, Xue-Bo Jin, Ting-Li Su, Zhen-Yun Tang, Fa-Fa Wang, Na Xiang, Jian-Lei Kong

**Affiliations:** 1 School of Computer Information and Engineering, Beijing Technology and Business University, Beijing 100048, China; yishenglun@st.btbu.edu.cn (S.-L.Y.); jinxuebo@btbu.edu.cn (X.-B.J.); wangfafa@st.btbu.edu.cn (F.-F.W.); xiangna@st.btbu.edu.cn (N.X.); kongjianlei@btbu.edu.cn (J.-L.K.); 2 Beijing Key Laboratory of Big Data Technology for Food Safety, Beijing Technology and Business University, Beijing 100048, China; 3 The Key Laboratory of Urban Security and Disaster Engineering, Ministry of Education, Beijing University of Technology, Beijing 100124, China; tzy@bjut.edu.cn

**Keywords:** online denoising, the second-order adaptive statistics model, Kalman filter, Yule–Walker algorithm, real-time data processing

## Abstract

Online denoising is motivated by real-time applications in the industrial process, where the data must be utilizable soon after it is collected. Since the noise in practical process is usually colored, it is quite a challenge for denoising techniques. In this paper, a novel online denoising method was proposed to achieve the processing of the practical measurement data with colored noise, and the characteristics of the colored noise were considered in the dynamic model via an adaptive parameter. The proposed method consists of two parts within a closed loop: the first one is to estimate the system state based on the second-order adaptive statistics model and the other is to update the adaptive parameter in the model using the Yule–Walker algorithm. Specifically, the state estimation process was implemented via the Kalman filter in a recursive way, and the online purpose was therefore attained. Experimental data in a reinforced concrete structure test was used to verify the effectiveness of the proposed method. Results show the proposed method not only dealt with the signals with colored noise, but also achieved a tradeoff between efficiency and accuracy.

## 1. Introduction

With recent improvements in sensor technologies, information networks, and telemetry, an enormous amount of data is collected every day. At the same time, with the help of data processing techniques, policy-makers and scientists are now able to deploy these sampled data in significant applications such as target location [1], disease case count prediction [2], structural health monitoring [3], and financial forecasting [4]. However, signals may be subject to random noise in practical processes, due to such reasons as incorrect measurements, faulty sensors, or imperfect data collection. Any noise and instability can be considered as the source of error, which would result in signal distortion.

How to eliminate the influence of the noise in measured data and extract the useful information has been a focus of information science research. Currently, existing algorithms primarily focus on the offline denoising problem, which requires a full set of data to accomplish the denoising process. Common solutions can be divided into two categories, i.e., offline denoising in the time domain [5,6,7] and in the frequency domain [8]. Specifically, in the time domain, Weissman et al. [5] proposed the discrete universal denoiser (DUDE) algorithm for offline denoising. DUDE assumes the statistical knowledge of the noise mechanism, but makes no assumptions on the distribution of the underlying data. Furthermore, Motta et al. [6] presented an extension of the DUDE specialized for the denoising of grayscale images. Moon et al. [7] introduced S-DUDE, a new algorithm for denoising discrete memoryless channel(DMC)-corrupted data. Aside from DUDE, the wavelet transform method is another classical offline method. Various offline denoising techniques based on the modified wavelet transform method can be found in different denoising-related applications [9,10,11]. The methods above possess strong capabilities for removing noise and preserving data details. However, the commonality is that they require all the data as a necessary condition.

In the frequency domain, Bhati et al. [12] designed a time–frequency localized three-band biorthogonal linear phase wavelet filter bank for epileptic seizure electroencephalograph (EEG) signal classification. For nonlinear and nonstationary signals, Gao et al. [13] proposed a novel framework of amalgamating empirical mode decomposition (EMD) with variable regularized two-dimensional sparse non-negative matrix factorization (v−SNMF2D) for single-channel source separation. The method can avoid certain strong constraints in separating blind source signals, and provide a robust sparse decomposition. However, it also needs all the data for the denoising process, and is not suitable for online purposes. Yin et al. [8] presented a novel approach for mechanical vibration signal denoising filter using partial differential equation(PDE) and its numerical solution. This method is not only easy to achieve but also can obtain smooth filtering results. However, this method has some limitations. First, the cut-off frequency in practical systems is difficult to determine. Second, as the amount of data increases, the arithmetic speed is slower, and the inverse matrix will be out of memory.

In real-world applications, such as condition monitoring [14], critical event forecasting [2] or health monitoring [3], data is susceptible to the noise and instability of the measurement process. Existence of serious noise in real-time data may cause not only the inaccurate outcomes but also the failure of the entire system. Meanwhile, in many physical systems, we need to collect and process data in real time. Therefore, online denoising is very necessary in data processing.

On one hand, smoothing and trend filtering methods are typically used in data processing to remove useless information. The moving average method is a well-known and popular technique due to its simplicity for online denoising [15] and long-term forecasting applications [16,17]. However, when there is too much random impulse disturbance in the experiment process, the measured data will show a strong maneuvering feature, and the smoothing filtering is not applicable to deal with it. In recent years, further study has improved these canonical methods. Exponential smoothing was used in prediction applications such as tourism demand [18], composite index data [19] or inflation rate [20]. Jere et al. [20] described the Holt’s exponential smoothing algorithm based on the assumption of a model consisting of a trend. Recent observations are expected to have significant influence on the future values in a series. Therefore, the prediction of the future value could be implemented through some previous points. Online denoising could also be achieved in a similar way. For instance, Goh et al. [21] proposed a sequential myriad smoothing approach for tracking a time-varying location parameter corrupted by impulsive symmetric α stable noise.

On the other hand, the Kalman filter, as a popular estimation method, has been widely used in various online applications, like navigation [22], target tracking [23], and signal processing [24], etc. The most important advantage is that it can not only retain the useful information but also obtain the most optimal estimate online. For example, the Kalman filter is used to minimize the error in stereo vision-based distance measurement data (3D position of pedestrians) in [25]. Huang et al. [26] developed the noise reduction method by a hybrid Kalman filter with an autoregressive moving average (ARMA) model. The coefficients of the AR model for the Kalman filter are calculated by solving for the minimum square error solutions. Rosa et al. [27] presented a Kalman filter-based approach for track reconstruction in a neutrino telescope, which can effectively remove the errors caused by noise and improve the accuracy of the data.

It needs to be pointed out that, when using the Kalman filter, an accurate system dynamic model would offer great help to achieve the optimal estimation. Miao et al. [28] used the Kalman filter with several different kinds of system models to remove the noise of the storage volume data of the internet center. Due to the difficulty in obtaining the density characteristic of the practical data, the adaptive model was proposed to capture the characteristics of the moving targets in [29], and estimate the acceleration based on the adaptive parameter.

In this paper, a denoising method for real-time data with unstable fluctuation and colored noise was investigated. For the sake of the data features and the online requirement, the Kalman filtering method based on a second-order adaptive statistics model was proposed here, and its performance was verified by some real test data. Moreover, the test data was processed via another two representative methods: first-order exponential smoothing [18] and Holt’s exponential smoothing [20], and the results demonstrated that the proposed method could give a better effect.

Compared to previous works, the contribution of this work is that we used a second-order adaptive model for online denoising, which can obtain a better denoising performance for the measurements in the reinforced concrete structure test experiment. The comparison between our model and the third-order model [29] is given in Section 3, and the results show that the developed second-order adaptive model here can obtain a smaller error and consume less time.

The structure of this paper is as follows. Section 2 presents the specific method of the second-order adaptive statistics model. The overview of the experiment is provided in Section 3. Section 4 discusses the robustness and the real-time performance. Some conclusions are given in Section 5.

## 2. Online Denoising Algorithm Based on Kalman Filtering and the Adaptive Statistics Model

For the purpose of removing the unexpected noise in an online mode, Kalman filtering was actually a competitive solution, where only the estimation derived in the previous step and the measurements in the current step were required to compute the new estimated values. However, this is not enough to obtain the desired results. A reasonable model that could describe the dynamic features of the data is another impact factor in the denoising process. Therefore, a second-order adaptive statistics model is presented later in this section, and the method to compute the adaptive parameter is explained in detail as well.

### 2.1. Online Denoising Algorithm Based on Kalman Filtering

Kalman filtering is one of the most classical recursive algorithms that gives the optimal estimation of the state vector. The Kalman filter estimates a process by using a form of feedback control: the filter estimates the process state at some time and then obtains feedback in the form of (noisy) measurements. As such, the equations for the Kalman filter fall into two groups: state update equation and measurement update equation, which can be expressed as:

(1) $$\begin{array}{c} x(k+1)=\Phi (k+1|k)x(k)+U(k)u(k)+w(k)\\ z(k+1)=H(k+1)x(k+1)+v(k+1) \end{array}$$

where *x* is the state vector of the system to be estimated, whose initial value and covariance are known as $x_{0}$ and $P_{0}$. Φ(k+1|k) is the state-transition matrix. u(k) is the system input and U(k) is the corresponding matrix. w(k) and v(k) are the process noise and measurement noise respectively, and the variance of v(k) is known (as *R*). Note that both w(k) and v(k) are white noise with zero mean and independent of the initial state $x_{0}$. z(k) is the measurement vector and H(k) is the observation matrix.

The Kalman filtering considers the correlation between errors in the prediction and the measurements. The algorithm is in a predict-correct form, which is convenient for implementation as follows:

(1) Initialization:
  (2) $$\hat{x}\left(0|0\right)=x_{0}\;\;\;\;\;\;P\left(0|0\right)=P_{0}\;$$
(2) Prediction:
  (3) $$\hat{x}\left(k+1|k\right)=\Phi \left(k+1|k\right)\hat{x}\left(k|k\right)+U\left(k\right)u\left(k\right)\;$$
  (4) $$P\left(k+1|k\right)=\Phi \left(k+1|k\right)P\left(k|k\right)\Phi^{T}\left(k+1,k\right)+Q\left(k\right)\;$$
(3) Correction:
  (5) $$K\left(k+1\right)=P\left(k+1|k\right)H^{T}\left(k+1\right){\left[H\left(k+1\right)P\left(k+1|k\right)H^{T}\left(k+1\right)+R\right]}^{T}\;$$
  (6) $$\hat{x}\left(k+1|k+1\right)=\hat{x}\left(k+1|k\right)+K\left(k+1\right)\left[y\left(k+1\right)-H\left(k+1\right)\hat{x}\left(k+1|k\right)\right]\;$$
  (7) P(k+1|k+1)=[I-K(k+1)H(k+1)]P(k+1|k)

According to the equations above. The algorithm works in a two-step process. In the prediction step, the Kalman filter produces estimates of the current state variables along with their uncertainties. Once the outcome of the next measurement (necessarily corrupted with some amount of error, including random noise) is observed, these estimates are updated using a weighted average, with more weight being given to estimates with higher certainty. Since the algorithm can run recursively, we can implement it step by step, that is, the denoised data can be obtained in real time.

### 2.2. Adaptive Statistics Model for Online Denoising

Considering the unstable fluctuation of the data and the existence of colored noise, the linear time-invariant model with noise as used in Section 2.1 may not be suitable for describing this kind of data. Therefore, we proposed a second-order adaptive statistics model to deal with these challenges. Let x, $\dot{x}$ be the data itself and the gradient, respectively. The state vector is expressed as $x={\left[x,\dot{x}\right]}^{T}$ throughout this paper unless stated otherwise explicitly.

Referring to the colored noise, it mainly lies in the changing process of the data gradient. When the data is varying with time, its gradient will follow certain rule: value of the gradient at the next time tick is always within the neighborhood of the current predicted gradient value. Therefore, the gradient can be computed as:

(8) $$\dot{x}\left(t\right)=\bar{g}\left(t\right)+\Delta \left(t\right)\;$$

where $\bar{g}\left(t\right)$ is the predicted value of $\dot{x}\left(t\right)$ in current interval. In particular, Δ(t) stands for the maneuvering change with colored noise. Considering that Kalman filter has specific requirements for the type of the noise, colored noise in Δ(t) needs to be processed. Therefore, the Wiener–Khinchin theorem was introduced here, which assumes it corresponds to the first-order stationary Markov process:

(9) $$\dot{\Delta }\left(t\right)=-\alpha \Delta \left(t\right)+w\left(t\right)$$

where α is the parameter of maneuvering frequency [29], and w(t) is a Gussian white noise with zero mean and a variance of $\sigma_{\Delta }^{2}$. With the two equations above, the change of the gradient can be written as:

(10) $$\ddot{x}\left(t\right)=-\alpha \dot{x}\left(t\right)+\alpha \bar{g}\left(t\right)+w\left(t\right)\;$$

since $\ddot{x}\left(t\right)=\dot{\Delta }\left(t\right)$ over any sampling interval.

Therefore, the state-space representation of the continuous-time adaptive model is:

(11) $$\dot{x}\left(t\right)=\left[\begin{array}{cc} 0 & 1\\ 0 & -\alpha \end{array}\right]x\left(t\right)+\left[\begin{array}{c} 0\\ \alpha \end{array}\right]\bar{g}\left(t\right)+\left[\begin{array}{c} 0\\ 1 \end{array}\right]w\left(t\right)\;$$

Let $A=\left[\begin{array}{cc} 0 & 1\\ 0 & -\alpha \end{array}\right]$, $B=\left[\begin{array}{c} 0\\ -\alpha \end{array}\right]$, $C=\left[\begin{array}{c} 0\\ 1 \end{array}\right]$. The solution of equations is:

(12) $$x\left(t\right)=e^{At}x\left(t_{0}\right)+\int_{0}^{t}e^{A(t-\lambda )}B\bar{g}\left(\lambda \right)d\lambda +\int_{0}^{t}e^{A(t-\lambda )}Cw\left(\lambda \right)d\lambda \;$$

We assume $t=t_{0}+T$ and $t_{0}=kT$. Then we can get the discrete-time equivalent as the following:

(13) $$x\left(k+1\right)=\Phi \left(k+1|k\right)x\left(k\right)+U\left(k\right)\bar{g}\left(k\right)+w\left(k\right)\;$$

With Laplace transforms, matrix Φ(k+1|k) can be expressed as:

(14) $$\Phi \left(k+1|k\right)=e^{AT}=\left[\begin{array}{cc} 1 & \frac{\left(1-e^{-\alpha T}\right)}{\alpha }\\ 0 & e^{-\alpha T} \end{array}\right]$$

Matrix U(k) can be described as:

(15) $$U\left(k\right)=\int_{0}^{T}e^{A(T-\lambda )}Bd\lambda =\left[\begin{array}{c} T\\ 1 \end{array}\right]-\left[\begin{array}{c} \frac{\left(1-e^{-\alpha T}\right)}{\alpha }\\ e^{-\alpha T} \end{array}\right]=\left[\begin{array}{c} T-\frac{1-e^{-\alpha T}}{\alpha }\\ 1-e^{-\alpha T} \end{array}\right].\;$$

The variance of the w(k) can be computed in the following way:

(16) $$Q\left(k\right)=E\left[w\left(k\right)w^{T}\left(k\right)\right]=\int_{0}^{T}e^{A(T-\lambda )}C\sigma_{\Delta }^{2}Ce^{A(T-\lambda )}d\lambda =2\alpha \sigma_{\Delta }^{2}\left[\begin{array}{cc} q_{11} & q_{12}\\ q_{12} & q_{22} \end{array}\right]$$

where

(17) $$\begin{array}{c} q_{11}=\frac{1}{2\alpha^{3}}\left(4e^{-\alpha T}-3-e^{-2\alpha T}+2\alpha T\right)\\ q_{12}=\frac{1}{2\alpha^{2}}\left(e^{-2\alpha T}+1-2\alpha T\right)\\ q_{22}=\frac{1}{2\alpha }\left(1-e^{-2\alpha T}\right) \end{array}\;$$

### 2.3. Adaptive Parameter Adjustment via the Yule-Walker Algorithm

In the previous subsection, a statistics model was presented to capture the fluctuation features in the measured data. It needs to be pointed out that in the proposed model, the adaptive parameter α is not only unknown, but also self-adaptive.

We adopted the following method to update parameter α and $\sigma_{\Delta }^{2}$ based on the Yule–Walker estimated algorithm [29]. First of all, we need to discretize the Equation (9). Through substituting *A* to -α and *C* to 1 in Equations (14) and (16), we can obtain its discrete-time equivalent:

(18) Δ(k)=βΔ(k-1)+w(k-1)

where w(k-1) is a discrete-time zero-mean white noise sequence with variance $\sigma_{\Delta \omega }^{2}=\sigma_{\Delta }^{2}\left(1-\beta^{2}\right)$ and $\beta =e^{-\alpha T}$. Then, the method of parametric update is as follows:

(1) Initialization:
  (19) $$\alpha \left(0\right)=\alpha_{0}\;\;\;\sigma_{\Delta }^{2}\left(0\right)=\sigma_{\Delta 0}^{2}\;\;\;r_{0}\left(0\right)={\dot{x}}_{0}\cdot {\dot{x}}_{0}\;\;\;r_{0}\left(1\right)={\dot{x}}_{0}\;$$
(2) Set the estimation of gradient and $\bar{g}\left(k\right)$ as:
  (20) $$\hat{\dot{x}}\left(k\right)=\dot{x}\left(k|k\right)\;$$
  (21) $$\bar{g}\left(k\right)=\frac{1}{k}\sum_{k=0}^{k}\hat{\dot{x}}\left(k|k\right)\;$$
  The parameter of σ(k) is satisfied with the first-order stationary Markov process:
  (22) $$\beta \left(k\right)=\frac{r_{k}\left(1\right)}{r_{k}\left(0\right)}\;\;\;\;\;\;\sigma_{\Delta \omega }^{2}\left(k\right)=r_{k}\left(0\right)-\alpha \left(k\right)r_{k}\left(1\right)\;$$
(3) Parameter update:
  (23) $$\begin{array}{c} r_{k}\left(1\right)=r_{k-1}\left(1\right)+\frac{1}{k}\left[\hat{\dot{x}}\left(k\right)\hat{\dot{x}}\left(k-1\right)-r_{k-1}\left(1\right)\right]\\ r_{k}\left(0\right)=r_{k-1}\left(0\right)+\frac{1}{k}\left[\hat{\dot{x}}\left(k\right)\hat{\dot{x}}\left(k\right)-r_{k-1}\left(0\right)\right] \end{array}\;$$
  and
  (24) $$\begin{array}{c} \alpha \left(k\right)=-\frac{\ln r_{k}\left(1\right)-\ln r_{k}\left(0\right)}{T}\\ \sigma_{\Delta }^{2}\left(k\right)=\frac{r_{k}\left(0\right)-\alpha \left(k\right)r_{k}\left(1\right)}{1-{\left(\frac{r_{k}\left(1\right)}{r_{k}\left(0\right)}\right)}^{2}} \end{array}$$

Then, we can use the Equation (24) to get α and $\sigma_{\Delta }^{2}$ so that we can achieve the purpose of updating the system parameters.

**Figure 1:**
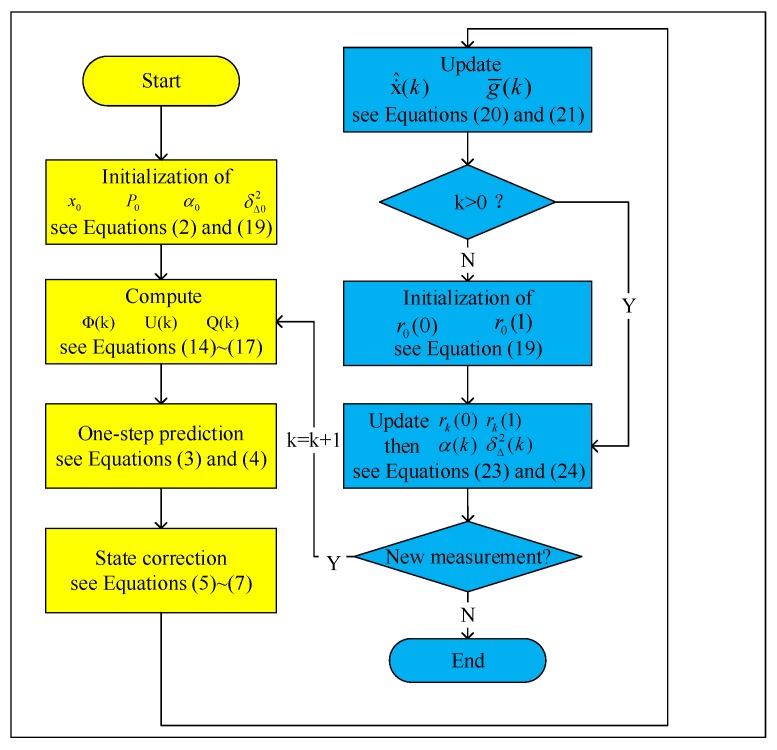
The flow chart of the proposed online denoising method.

Using the method described in this section, online denoising of data with unstable fluctuation and colored noise was then accomplished. The flow chart of the proposed method was given in Figure 1. It can be seen that the method consists of two parts within a closed loop. The first one is to estimate the system state with the Kalman filter based on the second-order adaptive statistics model, and the other is to update the adaptive parameter in the model by the Yule–Walker algorithm. In the next section, the effectiveness of this method will be evaluated via the experiment data from a reinforced concrete structure test, and the results will also be compared to some other representative online denoising methods.

## 3. Experiments

**Figure 2:**
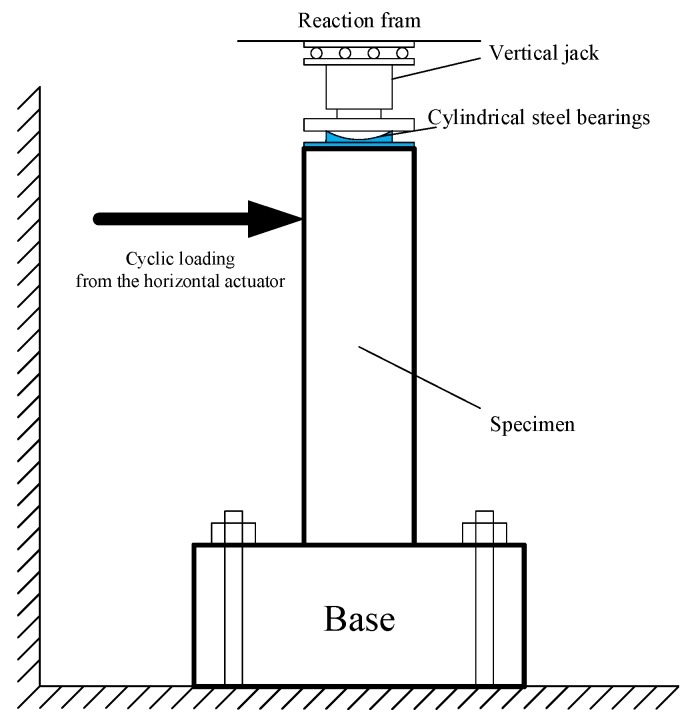
The configuration of the experiment.

In order to verify the effectiveness of the proposed algorithm, experimental data from the test of a reinforced concrete structure was adopted. The configuration of the experiment is shown in Figure 2. It was a quasi-static test for the column made by Chinese Grade 345 steel and C30 Grade concrete [30]. During the experiment, the column was tested under constant axial load and cyclic bending. Through this experiment, deformation displacement at different time samples were obtained, which correspond to the measurements in the proposed algorithm. Although the entire data was ready before denoising as well, the process was implemented in an ‘online’ mode, i.e., only the measurement of the ‘current’ sampling time and previous result would be used in computation. The necessity of the online mode for this background is because the actual value of the measured state has great effect on the identification of the structure security, and it needs to be known during the monitoring process. In this experiment, the sampling time was set as 0.001 s.

**Figure 3:**
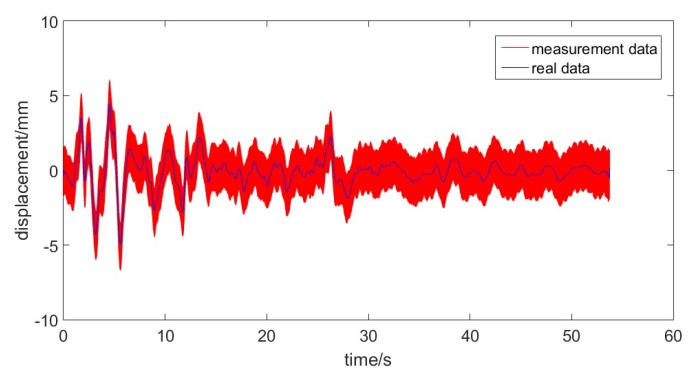
The real data and measurement data.

Figure 3 gives the measurement and the real data, which is used to test the performance of the developed method. The measurement data came from the experiment and the real data came from the offline filter with high degree of accuracy. As can be clearly seen from Figure 3, the measured data possessed a unstable fluctuation as well as the existence of the colored noise.

In this paper, we compared a second-order adaptive statistics model with various other methods such as first-order exponential filtering, Holt’s exponential filtering or a third-order adaptive statistics model to deal with the denoising problem for the real-time deformation displacement data. In order to evaluate these methods, mean and covariance of the error were compared. In addition, the root-mean-square error (RMSE) was used. The RMSE is very commonly used and makes for an excellent general purpose error metric for numerical predictions. Specifically, ‘mean’ here represents averaged absolute value of difference between the real data and the denoised data, i.e.,

(25) $$\mathrm{mean}=\frac{\sum_{i=1}^{n}\left|r_{i}-d_{i}\right|}{n}\;$$

where *n* is the number of the measurements, $r_{i}$ is the ith real data and $d_{i}$ is the corresponding denoised data.

Then, the covariance is defined as the following:

(26) $$\mathrm{cov}=\frac{\sum_{i=1}^{n}{\left(\mathrm{mean}-\left|r_{i}-d_{i}\right|\right)}^{2}}{n}$$

Finally, the RMSE can be expressed as the following:

(27) $$\mathrm{RMSE}=\sqrt{\frac{\sum_{i=1}^{n}{\left(d_{i}-r_{i}\right)}^{2}}{n}}\;$$

In the following context, three cases are implemented. In the first two cases the comparison between different denoising methods is depicted, while a third case is given to discuss the effect of the initial value on the denoising performance. In Section 3.1, the adaptive statistics models, including the second-order model and the third-order model, were used to deal with the data; in Section 3.2, we compared the developed method with the first-order exponential filtering and Holt’s exponential filtering, respectively; in Section 3.3, through eliminating data within the adjustment process and retaining the posterior convergent data, the denoising effect was obviously improved.

### 3.1. The Denoising Effect of the Adaptive Statistics Model

**Figure 4:**
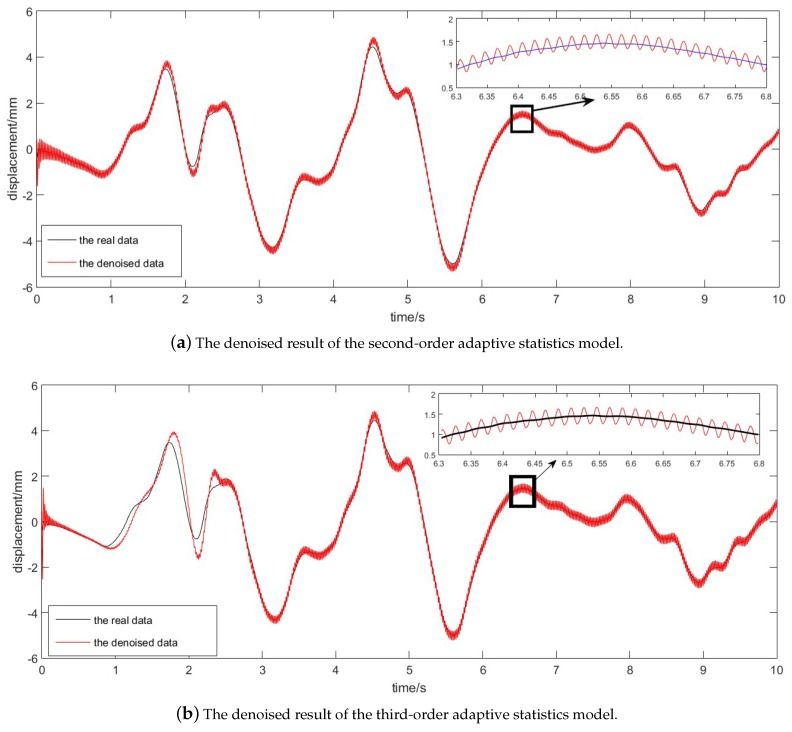
The denoised result of the adaptive statistics models.

The performances of the second-order and the third-order adaptive methods for online denoising were compared in this part. The denoised results are shown in Figure 4. Since the difference was too small, we provide a detailed part of the curves in the small picture, and 500 points from 6.3 s to 6.8 s are shown there. The results demonstrated that this algorithm is feasible and reliable with reasonable precision. Furthermore, through comparing the real data and the denoised data, the satisfactory denoising effect of the second-order adaptive statistics model was illustrated.

**Figure 5:**
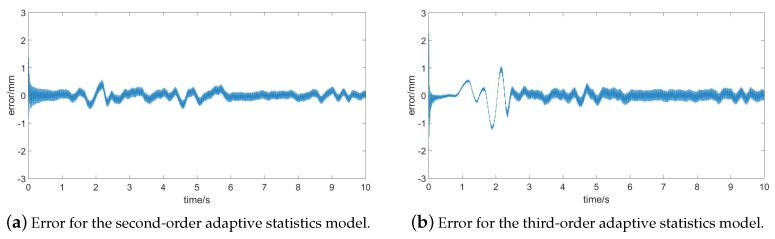
The error comparison of the adaptive statistics models for online denoising.

Comparing the second-order and third-order adaptive statistics models, we can find a satisfactory denoising effect in Figure 4a,b. However, from the result before 3 s, we might notice the third-order adaptive statistics model performs with poorer precision. Thus, the second-order adaptive statistics model can have advantages with respect to accuracy. Meanwhile, in order to better describe the error and compare the denoising precision, Figure 5 gives the error of the both models.

The results in Figure 5 show that the second-order adaptive statistics model has the smaller error. In order to better prove this conclusion, more groups of data were adopted to test the method, and each group contained 10,000 points. The symbol $mean_{m}$ here represents the mean of the whole data set. The results of the tests are shown in Table 1. Obviously, for each group, results from the second-order model all showed better performance both in mean, covariance and RMSE. As a whole, variance and RMSE of the second-order model was only about 0.0223 and 0.1461, respectively, better than that of the third-order model (0.1407 and 0.3129). On the other hand, Kalman filtering is an estimation algorithm which shows resemblance and proximity with the one-step prediction. We can estimate next step value by merely using the last measurement. Therefore, it is an online algorithm, that is, there is the negligible delay with the denoising process. In addition, the calculated amount of the second-order model is lower than for the third-order model. This is due to the more computational expense caused by the larger matrices in the higher-order model. Therefore, results showed the second-order adaptive statistics model could not only deal with the signals with colored noise in real time, but also achieve a tradeoff between efficiency and accuracy.

**Figure 6:**
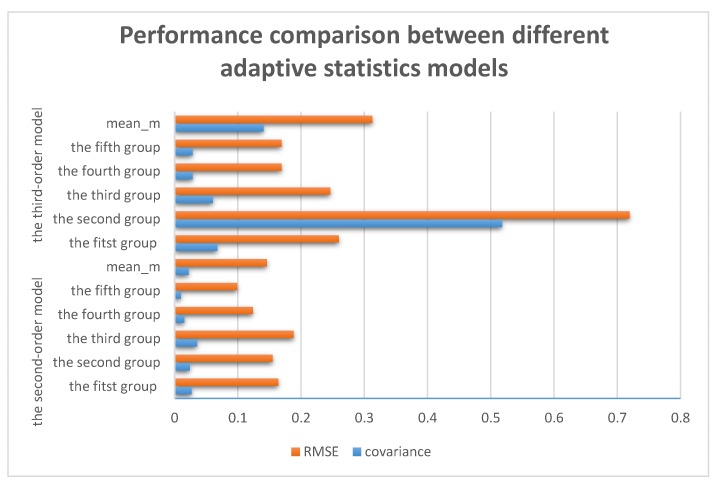
Covariance and RMSE of the adaptive statistics models.

Based on the results in the Table 1, it can be clearly seen that the second-order adaptive statistics model is better than the third-order one, because it provided better precision and faster speed in online denoising. Meanwhile, as we can see in Figure 6, more stable denoising effect and smaller RMSE can be offered by the second-order statistics model, in which the ‘orange column’ is the RMSE and the ‘blue column’ is the covariance for each group.

### 3.2. Comparison of the Denoising Effect between the Proposed Method and the Exponential Smoothing

Formerly, the exponential smoothing was typically for forecasting. Simultaneously, it could also be applied in online denoising [21]. When using the exponential smoothing, parameter selection is very important, as it can adjust the development tendency of the data trend. However, it is usually very subjective. Nowadays, the primary methods for parameters selection can be divided into two ways: one is the empirical method, the other is trial method. In this paper, we adopted the empirical method. Finally, we decided to utilize first-order exponential smoothing and Holt’s exponential smoothing for comparison with the result in Section 3.1.

#### 3.2.1. The Denoising Effect of the First-Order Exponential Smoothing

We utilized priori knowledge to select the parameters of 0.2, 0.5 and 0.8. A first-order exponential smoothing with different parameters was used to denoise the same five groups of data as those in Section 3.1, and the results are given in Table 2. According to those test results, we can draw a conclusion that first-order exponential smoothing [18] with a parameter of 0.2 possessed the best denoising effect.

#### 3.2.2. The Denoising Effect of the Holt’s Exponential Smoothing

Within Holt’s exponential smoothing [20], two kinds of states were usually used: one was the signal of the backward-smoothing, and the other was the tendency of the backward-smoothing. As a result, we introduced two parameters *a* and *b*. *b* was set to be 0.8 as empirical value, meanwhile, the parameter *a* was selected the same as the first-order exponential smoothing method, which was 0.2, 0.5 and 0.8. The same data was used as before, and the results are shown in Table 3.

It can be clearly seen in Table 3 that the best denoising effect can be acquired with the parameter *a* of 0.2 and *b* of 0.8, but the value of different indicators was still obviously larger than those of the proposed adaptive method. Table 4 gives a summary of performance comparison among different methods.

In these three categories of online denoising methods, the mean, covariance and RMSE of the adaptive statistics model are obviously the smallest. The results indicated that online denoising could be better achieved via the adaptive statistics model, because the system parameter could be adjusted dynamically as the denoising process was implemented. Furthermore, by contrasting the second-order adaptive model and the third-order adaptive model, we have come to the tentative conclusion that the effect of the second-order adaptive model is more outstanding. To sum up, between the two exponential smoothing methods, the Holt’s exponential smoothing with the parameter *a* of 0.2 and *b* of 0.8 has better denoising effect. However, among all the different methods conducted in this paper, the second-order adaptive statistics model presented the best performance. It not only showed good denoising accuracy, but also gave a faster processing speed.

### 3.3. The Effect of Initial Value on the Denoising Performance

**Figure 7:**
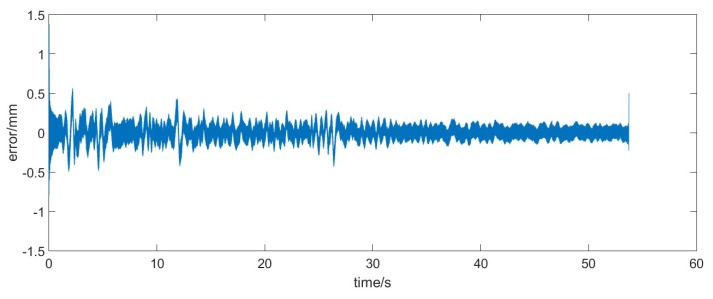
The real data and measurement data.

In this case, we would analyze the figure of the error data, as shown in Figure 7.

From the figure above it can be clearly seen that online denoising based on the adaptive statistics model had a regulatory process at the beginning. This is because the initial value of $x_{0}$ was zero and $P_{0}$ was very big. It thus appears that we could obtain the more precise filtering results through the index for selection. Actually, it needs to be emphasized that the convergence procedure existed in the adaptive model, that is, the denoising effect is be better as time goes on. Finally, we selected the last 5000 points to calculate the covariance and the mean.

**Figure 8:**
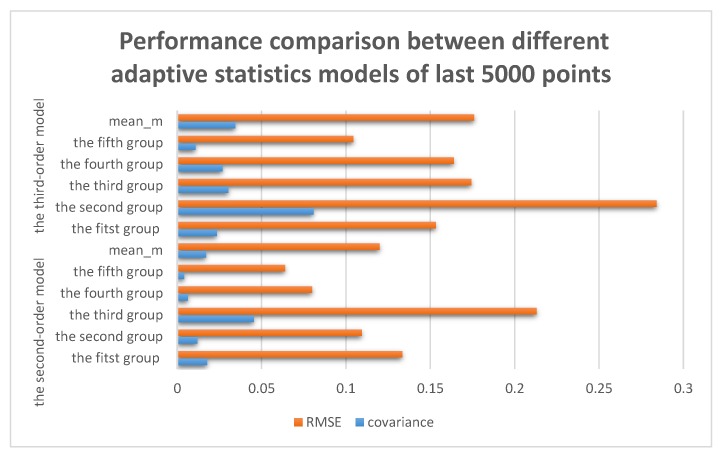
Covariance and RMSE of the adaptive statistics models of the last 5000 points.

As can be clearly seen from the Table 5 and Figure 8, mean, covariance and RMSE decreased significantly compared with those in Table 1 and Figure 6. By assessing the data, the covariance of the second-order model is only 0.0171 and RMSE is only 0.1200, while for the third-order model these values are 0.0345 and 0.1760, respectively. Recall that the best filter effect of exponential smoothing is about 0.2 and 0.43. This leads one to believe that the adaptive statistics model was superior to the exponential smoothing. When comparing two approaches using the adaptive statistics models, we can find the denoising effect of the second-order adaptive statistics model is better than the third-order adaptive statistics model. This is because the general trend of data seems more consistent with the second order.

In fact, except for precision, the second-order model has another preponderance. It possesses a smaller computation burden. We computed the runtime for each denoising process, and found the second-order adaptive model is faster than the smoothing filter and the third-order model. If we started to denoise the data with 52,741 counts, the elapsed time of the second-order model is 9.142300 s. On the contrary, we need 13.124500 s for the third-order model. Considering the statements above, we can come to the conclusion that the second-order adaptive statistics model is kind of more accurate and efficient method to proceed online denoising.

## 4. Discussion

In the previous section, through the experiment data and the comparison with other classical denoising methods, the effectiveness and superiority of the proposed method have been verified. In this part, we will focus on some other features of our denoising method, that is, the robustness and the real-time performance.

**Figure 9:**
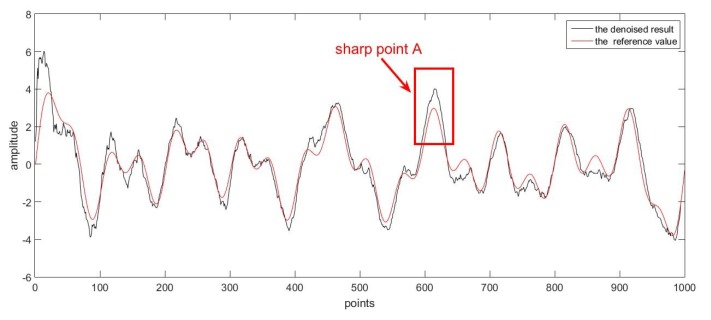
The denoised result and the reference value.

**Figure 10:**
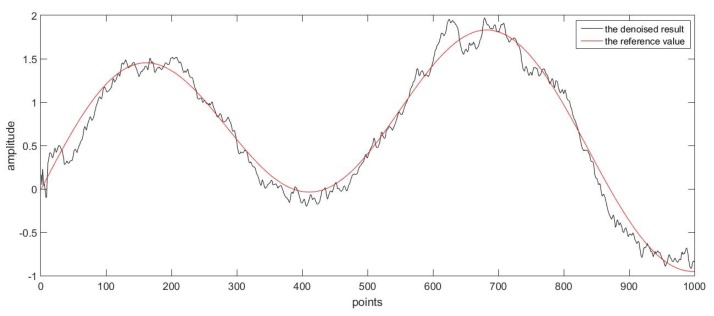
The reference value and the denoised result.

Firstly, as a good denoising method, it should be able to deal with various kinds of data. In order to prove this, two groups of superposed sinusoidal signals with colored noise were adopted. The sampling time for both groups was 0.001 s. The main difference between the two reference curves was that one had more sharp points while the other changed more gently, and the curves were shown respectively in Figure 9 and Figure 10 for comparison purpose.

**Figure 11:**
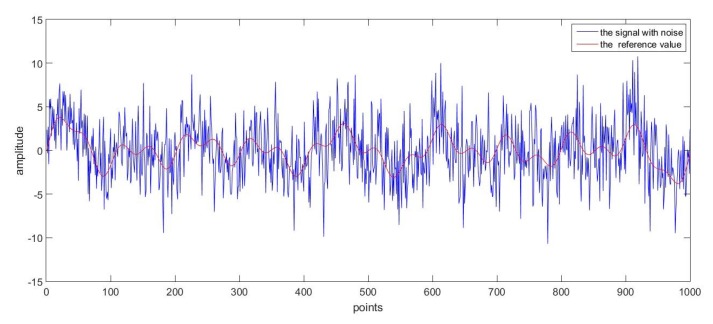
The signal with noise and the reference value.

The first group of data with noise is given in Figure 11, where the reference curve was totally drowned. With the proposed online denoising method, the estimated curve in Figure 9 could be derived. According to the comparison with the reference curve, the original noised signal was successfully processed.

**Figure 12:**
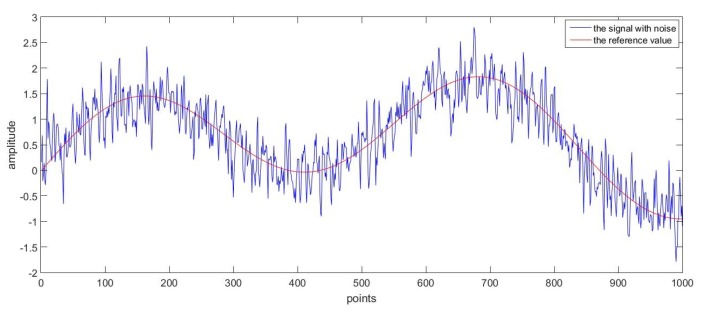
The reference value and the signal with noise.

For the second group of data with noise, as was shown in Figure 12, the denoising method was applied again. The difference between the denoised result and reference values was given in Figure 10. It can be seen in the figure that the overall trend of the curve was in good accordance with the reference values, and the oscillation was because some features of the noise was reserved due to a high-dimension process model.

Secondly, we would like to discuss the real-time performance of the proposed method. In order to achieve online denoising, the algorithm should have a fast processing speed. If not, latency would exist and might affect the result. As was stated before, the method proposed in this paper was based on Kalman filtering, which was a recursive algorithm. As long as the filtering process could finish before the new measurement was collected, the method was able to be implemented in real time. In the two simulations above, the time needed for one iteration was on average of 0.0003 s, which was far smaller than the sampling time of 0.001 s. It needs to be pointed out the difference like the subfigure shown in Figure 9 was not caused by the latency; it was mainly because of the sharp point A. The estimated points were changed with inertia, and they were then corrected to the measurement values by the recursive process. Therefore, this difference actually resulted from an estimation error other than the latency of the algorithm. In fact, the algorithm indeed performed in a real-time way as described above.

## 5. Conclusions

A huge amount of the real-time data is collected every second around the world. However, due to the imperfect measurement and data collection mechanisms, real-time data is distorted by various types of noise and instability. Therefore, working with noisy time series is an inevitable part of any real-time data processing task and must be addressed precisely. In the past decades, the demand for real-time data analysis techniques such as the first-order exponential smoothing and Holt’s exponential smoothing has grown dramatically. In this paper, we proposed an online denoising method for the real-time data with unstable fluctuation and colored noise.

This method consists of two parts within a closed loop. The first one is to estimate state based on the second-order adaptive statistics model. The other is to update the adaptive parameter in the model by the Yule–Walker algorithm. The effectiveness of method was demonstrated via an experiment, which not only processed the signals with colored noise, but also achieved a tradeoff between efficiency and accuracy. In addition, the performance of the proposed method was compared with some existing methods. Results showed that a more accurate and efficient denoising effect could be performed by employing the second-order adaptive statistics model with the Kalman filter for online denoising. 

## Figures and Tables

**Table 1:** Performance comparison between different adaptive statistics models.

	Second-Order Adaptive Statistics Model			Third-Order Adaptive Statistics Model		
	Mean/mm	Covariance	RMSE	Mean/mm	Covariance	RMSE
Firstgroup	0.1301	0.0269	0.1640	0.1762	0.0675	0.2598
Secondgroup	0.1130	0.0241	0.1552	0.4852	0.5179	0.7197
Thirdgroup	0.1375	0.0355	0.1884	0.1757	0.0607	0.2463
Fourthgroup	0.0930	0.0153	0.1237	0.1107	0.0287	0.1694
Fifthgroup	0.0721	0.0098	0.0990	0.1179	0.0286	0.1691
$Mean_{m}$	0.1091	0.0223	0.1461	0.2131	0.1407	0.3129

**Table 2:** Mean, covariance and RMSE of first-order exponential smoothing with different parameters.

Various Models	The Parameter of 0.2			The Parameter of 0.5			The Parameter of 0.8		
	Mean	Covariance	RMSE	Mean	Covariance	RMSE	Mean	Covariance	RMSE
Firstgroup	0.5864	0.4352	0.6597	0.9282	1.0813	1.0399	1.0112	1.2645	1.1245
Secondgroup	0.5867	0.4351	0.6596	0.9284	1.0814	1.0340	1.0113	1.2642	1.1244
Thirdgroup	0.5870	0.4353	0.6598	0.9287	1.0813	1.0399	1.0118	1.2637	1.1241
Fourthgroup	0.5861	0.4340	0.6588	0.9279	1.0794	1.0389	1.0108	1.2617	1.1233
Fifthgroup	0.5864	0.4340	0.6588	0.9282	1.0802	1.0393	1.0112	1.2625	1.1236
$Mean_{m}$	0.5865	0.4347	0.6593	0.9283	1.0807	1.0384	1.0113	1.2633	1.1240

**Table 3:** Mean, covariance and RMSE the of Holt’s exponential smoothing with different parameter *a*.

Various Models	The Parameter *a* of 0.2			The Parameter *a* of 0.5			The Parameter *a* of 0.8		
	Mean	Covariance	RMSE	Mean	Covariance	RMSE	Mean	Covariance	RMSE
Firstgroup	0.3832	0.1891	0.4349	0.5978	0.4679	0.6840	0.9326	1.0542	1.0267
Secondgroup	0.3824	0.1870	0.4324	0.5973	0.4669	0.6833	0.9329	1.0532	1.0262
Thirdgroup	0.3824	0.1870	0.4324	0.5975	0.4667	0.6832	0.9326	1.0532	1.0262
Fourthgroup	0.3816	0.1861	0.4314	0.5965	0.4655	0.6823	0.9321	1.0505	1.0249
Fifthgroup	0.3818	0.1857	0.4309	0.5968	0.4657	0.6824	0.9324	1.0512	1.0253
$Mean_{m}$	03823	0.1870	0.4324	0.5972	0.4665	0.6830	0.9325	1.0525	1.0259

**Table 4:** The results by several kinds of online denoising methods.

Various models	Various orders/parameters	${\mathrm{Mean}}_{m}$ of mean/mm	${\mathrm{Mean}}_{m}$ of covariance	${\mathrm{Mean}}_{m}$ of RMSE
Adaptive statistics model	Second-order	0.1091	0.0223	0.1461
	Third-order	0.2131	0.1407	0.3129
First-order exponential smoothing	Parameter of 0.2	0.5865	0.4347	0.6593
	Parameter of 0.5	0.9283	1.0807	1.0384
	Parameter of 0.8	1.0113	1.2633	1.1240
Holt’s exponential smoothing	Parameter *a* of 0.2	0.3823	0.1870	0.4324
	Parameter *a* of 0.5	0.5972	0.4665	0.6830
	Parameter *a* of 0.8	0.9325	1.0525	1.0259

**Table 5:** Mean, covariance and RMSE of the last 5000 points derived by the adaptive statistics model.

Various Model	Second-Order Adaptive Statistics Model			Third-Order Adaptive Statistics Model		
	Mean	Covariance	RMSE	Mean	Covariance	RMSE
Firstgroup	0.1106	0.0178	0.1334	0.1333	0.0235	0.1533
Secondgroup	0.0888	0.0120	0.1095	0.2301	0.0808	0.2842
Thirdgroup	0.1528	0.0454	0.2131	0.1343	0.0304	0.1743
Fourthgroup	0.0682	0.0064	0.0800	0.1260	0.0269	0.1640
Fifthgroup	0.0536	0.0041	0.0640	0.0842	0.0109	0.1044
$Mean_{m}$	0.0948	0.0171	0.1200	0.1416	0.0345	0.1760
